# Gestational Diabetes Mellitus and Postpartum Depressive Symptoms in Women with Low and Late Fertility

**DOI:** 10.3390/jpm15120609

**Published:** 2025-12-08

**Authors:** Vincenzo Zanardo, Gianluca Straface, Francesca Volpe, Agnese Suppiej, Tiziana Battistin

**Affiliations:** 1 Division of Perinatal Medicine, Abano Polyclinic, 35031 Abano Terme, Italy; gstraface@casacura.it (G.S.); fvolpe@casacura.it (F.V.); 2 Department of Medical Sciences, University of Ferrara, 44121 Ferrara, Italy; sppgns@unife.it; 3 Department of Neurosciences and Rehabilitation, Paediatrics Section, University of Ferrara, 44121 Ferrara, Italy; tiziana.battistin@unife.it

**Keywords:** Gestational diabetes mellitus, GDM, low and late fertility, mood disturbances, Edinburgh postnatal depression scale, EPDS, Anhedonia, Anxiety, and Depression subscales

## Abstract

**Background**: Dysregulation of the hypothalamic–pituitary–adrenal axis is implicated in both gestational diabetes mellitus (GDM) and mood disorders, suggesting a shared pathophysiology. However, the impact of GDM on maternal depressive symptoms, particularly among women with “low and late” fertility, remains poorly characterized. **Methods**: We compared the risk of postpartum depressive symptoms, assessed on the second postpartum day using the Edinburgh Postnatal Depression Scale (EPDS), with a cut-off score ≥ 12, and the Anhedonia, Anxiety, and Depression subscales, between Northeast Italian women with GDM and control participants with normal oral glucose tolerance tests (OGTT), classified as having “low and late fertility”. **Results**: Among the 2560 women included in the analysis, 231 (9.02%) had GDM. Compared with controls with normal OGTT, women with GDM were older (36.0 vs. 33.0 years, *p* < 0.001) and had higher pre-pregnancy BMI (23.4 vs. 21.6 kg/m^2^, *p* < 0.001), but lower gestational weight gain (GWG) (11.0 vs. 13.0 kg, *p* < 0.001), with no difference in parity [2.00 vs. 1.00, *p* = 0.5. In addition, GDM was not associated with increased postpartum depressive symptoms (15% EPDS scores ≥12 in both groups, *p* > 0.9) or with Anhedonia, Anxiety, or Depression subscale scores (*p* = 0.7). **Conclusions**: Advanced maternal age and reduced parity, hallmarks of women postponing childbearing, do not appear to confer an increased risk of early postpartum mood comorbidities in the context of GDM. Promoting healthy physical and mental well-being among women in this demographic category requires integrated strategies encompassing nutrition, healthcare, and education.

## 1. Introduction

Gestational diabetes mellitus (GDM) is one of the most common medical complications of pregnancy [1], with a global prevalence between 1% and 14% [2], including 10% of pregnancies in Italy [3]. GDM is a multifactorial condition arising from complex interactions between hormonal, metabolic, and genetic influences leading to hyperglycemia [4]. Additional factors, including pre-pregnancy obesity, excessive gestational weight gain (GWG), advanced maternal age, a family history of diabetes, and genetic predisposition, may further exacerbate metabolic dysregulation. The interplay of these determinants not only increases the risk of GDM but may also influence its severity, clinical course, and associated short- and long-term complications for both mother and child [5]. The negative physical health consequences of GDM are multifaceted, affecting both the mother and the infant during pregnancy, childbirth, and the postpartum period. Maternal hyperglycemia in GDM increases the risk of fetal macrosomia; obstetric complications such as shoulder dystocia, caesarean delivery, and perineal trauma; and neonatal complications, particularly perinatal asphyxia and hypoglycemia, the latter of which may result in long-term neurological impairment if left untreated [6,7].

Much less is known about the potential impact of GDM on maternal mental health, especially the risk of mood disorders. This is particularly relevant given the growing body of evidence implicating dysregulation of the hypothalamic–pituitary–adrenal (HPA) axis in both GDM and depressive disorders, thereby supporting a shared pathophysiological basis for their co-occurrence and progression [8]. Notably, the potential impact of GDM on maternal mental health, especially the risk of mood disorders, remains poorly characterized, and no consensus has been reached regarding the comorbidity of GDM and mood disorders. Some studies report that GDM does not increase the risk of prenatal or postnatal depression [9]. In contrast, other research has demonstrated a positive correlation between maternal age and the prevalence of anxiety and depression among women with GDM, with rates of these mood disorders increasing progressively with age [10,11]. Parallel epidemiological evidence indicates that the incidence of GDM itself rises with advancing maternal age [6]. This relationship is particularly relevant in light of the steady increase in maternal age at childbirth over the past four decades, a trend largely driven by social, economic, and lifestyle factors. In high-income countries, women are increasingly postponing childbearing, often due to prolonged education, career advancement, and financial planning, resulting in a compressed reproductive window and contributing to overall declines in fertility rates [12]. The convergence of these demographic and biological factors suggests that advanced maternal age may act as a shared determinant for both GDM and postpartum mood disorders, underscoring the need for age-specific preventive and screening strategies. This postponement of childbearing, a complex and multifaceted phenomenon often driven by career ambitions, economic considerations, and societal expectations, has contributed to the emergence of the so-called “low and late fertility” category [13]. 

Addressing these demographic challenges requires comprehensive policy approaches considering economic, social, and cultural factors influencing family planning decisions. Additionally, exploring the occurrence of postpartum depressive disorder is a vital next step in providing proper care. Therefore, the objective of this study was to examine the association between GDM and postpartum depressive symptoms, assessed using the Edinburgh Postnatal Depression Scale (EPDS) with its Anhedonia, Anxiety, and Depression subscales [14,15], in a low-risk, clinic-based sample of Northeast Italian women classified in the “low and late fertility” category, in line with prevalent demographic trends.

## 2. Materials and Methods

This study examined the association between GDM and early postpartum depressive symptoms, assessed using the EPDS, in a low-risk, clinic-based sample of high-income women characterized by low fertility rates and delayed childbearing. The study was conducted at the Abano Polyclinic in Abano Terme, located in an industrialized region of Northeast Italy, which supports approximately 1000 births per year. The patient population primarily comprised women with low and delayed fertility (parity: median 1.00, IQR 1.00–2.00; age: median 33.0 years, IQR 30.0–37.0), most of whom were married or cohabiting (92%), employed (73%), had advanced education (high school: 47%; bachelor’s: 14%; master’s: 29%), and experienced relatively high caesarean section rates (elective: 11%; emergency: 10%) [12].

All women presenting at term of gestation at the maternity ward of the hospital between January 2020 to August 2023 were screened for inclusion in the study, in accordance with the principles set forth in the 1964 Helsinki Declaration and its later amendments or comparable ethical standards. Pregnant women were first given information about the study at the antenatal healthcare centre and again at pre-discharge when they gave written informed consent to fill out the EPDS questionnaire. Permission to conduct this analysis was granted by the Institutional Ethic Review Board Committee of the Abano Polyclinic prior to its commencement (Ref. N. 24/2018; 9 November 2018). Exclusion criteria were an unsuccessful delivery at term, fetal anomalies, the presence of and/or being under treatment for prepartum psychological problems, and mothers not able to sufficiently read and understand Italian. 

**Figure 1:**
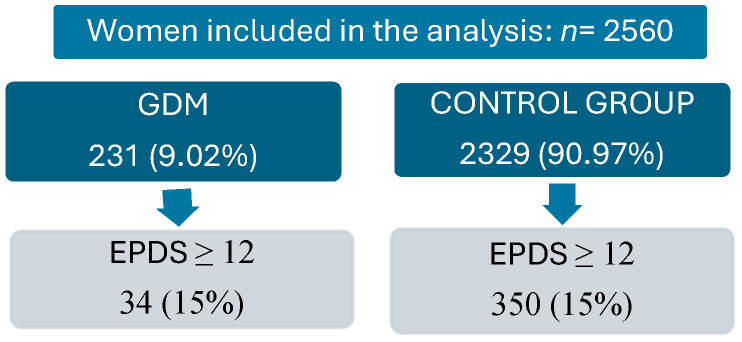
Flow chart of the study population.

Women in the final sample satisfied the clinical diagnostic criteria for GDM, as defined by the American Diabetes Association (ADA) in 2014 [16], and met the standard individual prenatal pathway for a low-risk pregnancy based primarily on the 2010 ISS-SNLG “Physiological pregnancy. Guideline.” [17], which typically consists of a multidisciplinary team of midwives, obstetricians, and, when needed, psychologists, family doctors, social workers, and other specialists (Figure 1).

Italian low-risk pregnancies are managed with a minimum of four structured visits, but most women attend 6–10 appointments spaced roughly monthly in the first two trimesters and more frequently as term approaches. Each visit includes a physical exam and education to address pregnancy-related issues (e.g., nutrition, weight gain, preventive measures during pregnancy, and safety).

Standard procedures at the hospital for uncomplicated pregnancies, both prior to and during the study period, included two-step delivery, delayed cord clamping following the onset of a vigorous neonatal cry, and brief hospitalization, all in accordance with established clinical policies and routines. Immediately after birth, all healthy newborns were placed skin-to-skin on their mothers’ chests, where they remained until either the first breastfeeding was successfully initiated or the newborn fell asleep. In most cases, the mother’s partner was present during and after childbirth. Skin-to-skin contact and frequent, on-demand breastfeeding were strongly encouraged. To foster a homelike atmosphere and support parent–newborn bonding, maternity rooms were designed to provide a sense of privacy and comfort. Throughout their stay, newborns roomed in with their mothers, who were encouraged to feed on demand. If breast milk intake was deemed insufficient, complementary formula feeding was provided. In the absence of obstetric complications, the standard length of stay was 48 h following both vaginal and caesarean deliveries. 

Women voluntarily completed the EPDS in person, provided by a healthcare professional, before discharge on the second day postpartum. EPDS is a self-administered questionnaire composed of 10 items scored on a 4-point Likert scale (0–3) designed to screen for post-partum depression symptoms. Postpartum depression represents the end of a continuum regarding the severity of symptoms. We stratified our study cohort into total EPDS score ≥ 12, respectively, as this cut-off categorisation and Anhedonia, Depression, and Anxiety subscale scores have been shown to be predictive of a particularly high risk of major depressive symptomatology [14,15]. 

Women who had GDM (GDM group) and those with normal OGTT results (Control group) were identified for comparison purposes. Sociodemographic data included maternal age, marital status, educational level, habits, occupation, diet, obstetric features (parity, age, height, pre-pregnancy BMI, GWG, gestational age), and obstetric characteristics, such as vaginal delivery and elective or emergency caesarean section. These and additional data regarding neonatal outcomes (birth weight) and feeding modalities at discharge, defined as exclusive breastfeeding, complementary feeding (breast milk with additional formula), and formula feeding according to WHO, were collected from medical records.

Descriptive statistics were reported as the median (interquartile range, IQR) for continuous variables and absolute numbers (percentages) for categorical variables. To compare the risk of postpartum depressive symptoms between participants with GDM and control participants with normal OGTT, the EPDS (cut-off score > 12) and its Anhedonia, Anxiety, and Depression subscales were used. Statistical analyses were performed with Pearson’s chi-square test to examine associations between categorical variables, while Fisher’s exact test was applied for comparisons in cases of small sample sizes. For continuous variables, group medians were compared using the Wilcoxon rank sum test. Benjamini–Hochberg correction was performed to account for the multiplicity of testing, controlling for the false discovery rate (FDR). Results were reported as *p*-values (statistical significance at *p* < 0.05). Statistical analyses were carried out using R software, version 4.4.0 (R Core Team 2023).

## 3. Results

Among the total number of women screened for inclusion in this study, 2560 women were included in this analysis, 231 (9.02%) had GDM (GDM group), and 2329 had normal OGTT (Control group). Baseline data and the clinical features of women with GDM and women in the control group with low and delayed fertility are reported in Table 1. 

The age of women in the GDM group was significantly higher than that in the Control group [36.0 (31.5–39.0) vs. 33.0 (30.0–37.0), *p* < 0.001], whereas the parity was comparable between groups [(2.00 (1.00–2.00) vs. 1.00 (1.00–2.00) *p* = 0.5]. Women in the GDM group also delivered significantly earlier [273 (270–280) vs. 278 (273–284) days, *p* < 0.01], but this was irrespective of delivery mode (*p* = 0.14). Educational level, diets, habits, marital status, occupation, and neonatal feeding at discharge were also shared/comparable between groups (*p* > 0.05). 

Pre-pregnancy BMI and GWG features of women with GDM and women in the Control group with low and delayed fertility are reported in Table 2.

Additionally, 8.71% of participants entered pregnancy underweight, 71.48% were of a healthy weight, 14.60% were overweight, and 5.19% were obese, with smoking (8.67%) and alcohol (10.9%) use during pregnancy being notably elevated. In addition, the pre-pregnancy BMI was significantly higher in GDM group women [23.4 (20.5–27.3) vs. 21.6 (19.8–23.9) kg, <0.001]. At delivery, maternal weight was similar between the GDM and Control groups, resulting in a significantly lower GWG in women with GDM [11.0 (8.5–14.0) vs. 13.0 (I11.0–16.0) kg, *p* <0.001]. The contribution of BMI categories to the occurrence of adequate, inadequate, or excessive gestational weight gain (GWG), based on the 2009 Institute of Medicine GWG guidelines [18], was as follows: among women with GDM, 94 (41%) had adequate GWG, 82 (35%) had inadequate GWG, and 55 (24%) had excessive GWG; in the control group, 1117 (47.30%) had adequate GWG, 539 (23%) had inadequate GWG, and 673 (29%) had excessive GWG (*p* < 0.001). Finally, there was no significant difference in neonatal body weight [3400 (3080–3640) vs. 3360 (3100–3640) g, *p* > 0.09] between the GDM and Control offspring groups. 

EPDS and Anhedonia, Anxiety and Depression subscale scores between the GDM and Control group women are presented in Table 3.

At discharge, on the second day postpartum, EPDS scores were comparable between GDM and Control group women [6.0 (4.0–9.5)] vs. 7.0 (4.0–10.0), *p* = 0.7]. Among these, 34 (15%) with GDM and 350 (15%) in the Control group with EPDS scores above the clinical cut-off of ≥12 were similarly diagnosed as at risk of depressive disorder (*p* > 0.09). Concurrently, no significant differences were observed in the scores for the EPDS subscales for Anhedonia [0.00 (0.00–0.50) vs, 0.00 (0.00–0.50), *p* = 0.7], Anxiety [1.00 (0.75–1.50) vs. 1.25 (0.75–1.75), *p* = 0.7], and Depression [0.25 (0.00–0.75) vs. 0.25 (0.00–0.75), *p* = 0.7]. 

## 4. Discussion

Examining the association between GDM and early postpartum depressive symptoms, assessed with the EPDS, in a low-risk, clinic-based sample of Northeast Italian women, we found that advanced maternal age and reduced parity, hallmarks of the low and late fertility group, did not confer an increased risk of early postpartum mood comorbidities in the context of GDM.

To our knowledge, no previous studies have specifically examined the association between GDM and postpartum depressive symptoms in women experiencing delayed first childbirth, treated as a single, bidirectional category reflective of a shortened reproductive window. We found that advanced maternal age and reduced parity, hallmarks of women postponing childbearing, do not appear to confer an increased risk of early postpartum mood comorbidities in the context of GDM. This finding highlights the importance of promoting healthy physical and mental well-being in this demographic through integrated strategies that encompass nutrition, healthcare, and education. In developed countries, delayed childbearing, often linked to career, economic, and social factors, has led to the growing “low and late fertility” group. Within this context, the prevalence of GDM and elevated depressive symptoms (EPDS > 12) is aligned with national averages [3,19]. Women with GDM were significantly older and had higher pre-pregnancy BMI than controls, but by delivery their weight was comparable, reflecting significantly lower GWG. Given that maternal age and parity are non-modifiable risk factors within a shortened reproductive window, we considered it more appropriate to classify women into a single “low and late fertility” category, rather than treating low fertility and delayed childbearing as separate yet concurrent challenges for GDM and postpartum depressive symptoms. Within this framework, the present population-based study highlights three key clinical characteristics that may serve as potential moderators of maternal depressive symptom risk: namely, classification within the “low and late fertility” category, the role of gestational weight management, and access to a multidisciplinary healthcare team offering integrated support in areas such as pregnancy-related care, nutrition, weight control, preventive strategies, and maternal safety. 

Delayed childbearing, typically defined as conceiving at or after the age of 31, has become increasingly common [20]. The average age at which women had their first child increased from 26.5 years in 2000 to 29.5 years in 2022 across Organization for Economic Co-operation and Development (OECD) countries, supported by complex and often contradictory interactions with fertility behaviours, as extensively discussed in the existing literature [21]. These dynamics are central to the Easterlin income–fertility paradox, first described in the 1970s, which noted that higher individual or household income is typically associated with lower fertility within countries [22]. Accordingly, Italy’s fertility rate is among the lowest in the OECD, with a total fertility rate of 1.2 children per woman in 2023 [21]. This trend is not unique to Italy; many developed countries, such as Spain, with 1.2 children per woman, and particularly Korea, with an estimated 0.7 children per woman in 2023, are experiencing similar, multifaceted declines in fertility rates, involving biological, socioeconomic, and behavioural factors [23]. In particular, this highlights the complex and often contradictory interactions among childbearing population characteristics, socioeconomic conditions, and fertility behaviours, as extensively discussed in the existing literature. This delay in starting families contributes bidirectionally to the overall decline in fertility rates, as the window for childbearing narrows with age [23]. 

Why this occurs is complex and not completely understood. Therefore, the rationale for studying the comorbidity of GDM and postpartum depressive symptoms partly relies on biological mechanisms supporting a healthy pre-pregnancy BMI and an adequate GWG at delivery [24]. Obesity is classified as a chronic disease, so it should be recommended to normalize weight before pregnancy and to prevent excessive GWG in order to prevent or mitigate adverse feto-maternal outcomes [25]. This is relevant considering that in high-income women, fertility postponement often coincides with their peak career years, when weight control habits are shaped by social, psychological, and cultural pressures of thinness, heightened body image concerns, and esthetic-driven physical activity all contribute. Urban professional norms and higher education levels reinforce slimness and health awareness, while access to cosmetic and weight-loss interventions may unintentionally promote underweight status and aversion to obesity, possibly imposing significant dietary restrictions and lifestyle changes during pregnancy [26]. Excessive and inadequate GWG have been associated with a higher risk of postpartum depressive symptoms [27]. Excessive GWG may contribute to poor body image, hormonal imbalance, and inflammation, while inadequate GWG can reflect nutritional deficiencies and stress, with both conditions potentially disrupting maternal mood regulation and increasing vulnerability to depression after childbirth [26].

However, the findings of this study may carry some clinical significance and suggest important implications for both practice and policy. They highlight the complex and often contradictory interactions among childbearing population characteristics, socioeconomic conditions, and fertility behaviours, as extensively discussed in the existing literature. Considering that pregnancy is often viewed as a period in which women are highly motivated to improve their health behaviours, the National Academy of Medicine developed recommendations for appropriate GWG based on pre-pregnancy BMI [18]. Guided by the welfare of the developing fetus, pregnant women with GDM, despite a higher pre-pregnancy BMI, significantly reduced their gestational weight gain thanks to integrated support and free access to a multidisciplinary healthcare team and counselling on key pregnancy-related topics such as nutrition, weight management, preventive care, and safety. With this comprehensive support, approximately one in four GDM women exhibited inadequate GWG, a proportion similar to that observed in the Control group for adequate GWG. This pattern may reflect the influence of social, economic, and lifestyle factors frequently associated with high-income populations, such as greater access to nutritional counselling, higher health literacy, structured prenatal care, and stronger adherence to weight management recommendations. Notably, this level of GWG management may have contributed to moderating the risk of postpartum depressive symptoms. 

A more nuanced analysis is needed to clarify whether GDM is an independent risk factor or merely associated with postpartum depressive symptoms through shared sociodemographic and clinical characteristics of women with low and late fertility [28]. Understanding these trends is critical for public health interventions, and coping with these trends requires integrated strategies in nutrition, healthcare, and education. Addressing these demographic challenges require comprehensive policy approaches that consider metabolic phenotype, psychosocial profile, and cultural factors influencing family planning decisions. Additionally, exploring the occurrence of postpartum depressive symptoms is a vital next step in providing proper care. 

We acknowledge that there are several limitations to this study. First, it was not designed to assess possible changes in clinical practice. Second, this is a retrospective study conducted at a single health centre. Maternal weight and height were self-reported, which may have affected the accuracy of the results. Lastly, as an observational study, it cannot definitively establish causal relationships. However, the findings remain credible, as the demographic, clinical, and baseline characteristics of the study sample accurately reflect those of the population being assessed.

## 5. Conclusions

Our research indicates that advanced maternal age and reduced parity, hallmarks of low-fertility populations, particularly among educated women in high-income societies, do not confer an increased risk of early postpartum mood comorbidities in the context of GDM. Tailoring prenatal care to the local epidemiological context, not only in terms of preventive nutritional counselling and lifestyle interventions but also in terms of monitoring and managing GWG, can help to mitigate adverse outcomes related to both maternal physical and mental health. Addressing these demographic challenges requires comprehensive policy approaches that consider the economic, social, and cultural factors influencing family planning decisions. Additionally, exploring the occurrence of postpartum depressive symptoms is a vital next step in providing proper care.

## Figures and Tables

**Table 1:** Baseline data of GDM and Control group women with ‘low and late’ fertility.

	GDM Group	Control Group	
Women, *n*	231 (9.02%) ^a^	2329 (90.97%) ^a^	Adjusted *p*-value ^b^
Age, years	36.0 (31.5–39.0)	33.0 (30.0–37.0)	<0.001
Gestational age, weeks	39.0 (38.6–40)	39.7 (39–40)	<0.001
Delivery mode:			0.14
Labour induction	14 (6.1%)	110 (4.7%)	
Vaginal	156 (68%)	1731 (74%)	
Elective caesarean section	38 (16%)	249 (11%)	
Emergency caesarean section	23 (10.0%)	239 (10%)	
Diets:			0.9
Mediterranean	220 (95%)	2239 (96%)	
Vegetarian	4 (1.7%)	35 (1.5%)	
Vegan	0 (0%)	3 (0.1%)	
Other	7 (3.0%)	52 (2.2%)	
Parity, %	2.00 (1.00–2.00)	1.00 (1.00–2.00)	0.5
Parity, *n*:			0.7
1	115 (50%)	1259 (54%)	
2	90 (39%)	852 (37%)	
3	26 (11%)	217 (9.3%)	
4	0 (0%)	1 (<0.1%)	
Educational level:			0.9
Secondary school	26 (11%)	214 (9.2%)	
High school	111 (48%)	1095 (47%)	
Three-year degree	28 (12%)	338 (15%)	
Master’s degree	66 (29%)	682 (29%)	
Smoking, *n*	24 (10%)	198 (8.5%)	0.6
Alcohol, *n*	29 (13%)	226 (9.7%)	0.4
Marital status:			>0.9
Single	1 (0.4%)	17 (0.7%)	
Married	119 (52%)	1267 (54%)	
Cohabiting	93 (40%)	877 (38%)	
Other	18 (7.8%)	168 (7.2%)	
Occupation:			>0.9
Housewife	18 (7.8%)	185 (7.9%)	
Student	31 (13%)	280 (12%)	
Unemployed	15 (6.5%)	155 (6.7%)	
Employed	167 (72%)	1709 (73%)	
Feeding at discharge:			0.4
Exclusive	152 (66%)	1670 (72%)	
Complementary	70 (30%)	573 (25%)	
formula	9 (3.9%)	86 (3.7%)	

^a^ Median (IQR); *n* (%); ^b^ Wilcoxon rank sum test; Fisher’s exact test; Pearson’s chi-squared test; false discovery rate correction for multiple testing.

**Table 2:** Pre-pregnancy BMI and GWG features of GDM and Control group women with ‘low and late fertility’.

	GDM Group	Control Group	
Women, *n*	231 ^a^	2329 ^a^	Adjusted *p*-value ^b^
Pre-pregnancy weight, kg	63 (56–73)	59 (54–66)	<0.001
Height, cm	164 (160–70)	165 (160–70)	0.024
Pre-pregnancy BMI	23.4 (20.5–27.3)	21.6 (19.8–23.9)	<0.001
Underweight	12 (5.2%)	211 (9.1%)	
Normal weight	133 (58%)	1697 (73%)	
Overweight	53 (23%)	321 (14%)	
Obese	33 (14%)	100 (4.3%)	
Weight at delivery, kg	75 (67–85)	73 (68–81)	0.2
GWG, kg	11.0 (8.5–4.0)	13.0 (11.0–6.0)	<0.001
GWG			<0.001
Adequate	94 (41%)	1117 (48%)	
Excessive	55 (24%)	673 (29%)	
Inadequate	82 (35%)	539 (23%)	
Neonatal birth weight, g	3400 (3080–3640)	3360 (3100–3640)	>0.9

GWG, gestational weight gain, according to the 2009 Institute of Medicine GWG guidelines; ^a^ Median (IQR); *n* (%); ^b^ Wilcoxon rank sum test; Fisher’s exact test; Pearson’s chi-squared test; false discovery rate correction for multiple testing.

**Table 3:** EPDS and Anhedonia, Anxiety and Depression subscale scores of gestational diabetes mellitus (GDM) and Control group women with low and late fertility.

	GDM Group	Control Group	
Women, *n*	*n* = 231 ^a^	*n* = 2329 ^a^	Adjusted *p*-value ^b^
EPDS total score	6.0 (4.0–9.5)	7.0 (4.0–10.0)	0.7
EPDS total scores > 12:			
No	197 (85%)	1979 (85%)	0.8
Yes	34 (15%)	350 (15%)	>0.9
EPDS subscales:			
Depression	0.25 (0.00–0.75)	0.25 (0.00–0.75)	0.7
Anhedonia	0.00 (0.00–0.50)	0.00 (0.00–0.50)	0.7
Anxiety	1.00 (0.75–1.50)	1.25 (0.75–1.75)	0.7
Depression	0.25 (0.00–0.75)	0.25 (0.00–0.75)	0.7

GDM, gestational diabetes mellitus; EPDS, Edinburgh Postnatal Depression Scale; ^a^ Median (IQR); *n* (%); ^b^ Wilcoxon rank sum test; false discovery rate correction for multiple testing.

## Data Availability

The original contributions presented in this study are included in the article. Further inquiries can be directed to the corresponding author.
